# Extracorporeal albumin dialysis in critically ill patients with liver failure: Comparison of four different devices—A retrospective analysis

**DOI:** 10.1177/03913988231191952

**Published:** 2023-08-23

**Authors:** Oliver Sommerfeld, Caroline Neumann, Jan Becker, Christian von Loeffelholz, Johannes Roth, Andreas Kortgen, Michael Bauer, Christoph Sponholz

**Affiliations:** Department of Anaesthesiology and Critical Care Medicine, Friedrich-Schiller-University Jena, Jena University Hospital, Jena, Thuringia, Germany

**Keywords:** Acute liver failure (ALF), acute-on-chronic liver failure (ACLF), albumin dialysis, extracorporeal liver support, renal-replacement therapy, apheresis and detoxification techniques

## Abstract

**Background:**

Besides standard medical therapy and critical care monitoring, extracorporeal liver support may provide a therapeutic option in patients with liver failure. However, little is known about detoxification capabilities, efficacy, and efficiency among different devices.

**Methods:**

Retrospective single-center analysis of patients treated with extracorporeal albumin dialysis. Generalized Estimating Equations with robust variance estimator were used to account for repeated measurements of several cycles and devices per patient.

**Results:**

Between 2015 and 2021 *n* = 341 cycles in *n* = 96 patients were eligible for evaluation, thereof *n* = 54 (15.8%) treatments with Molecular Adsorbent Recirculating System, *n* = 64 (18.7%) with OpenAlbumin, *n* = 167 (48.8%) Advanced Organ Support treatments, and *n* = 56 (16.4%) using Single Pass Albumin Dialysis. Albumin dialysis resulted in significant bilirubin reduction without differences between the devices. However, ammonia levels only declined significantly in ADVOS and OPAL. First ECAD cycle was associated with highest percentage reduction in serum bilirubin. With the exception of SPAD all devices were able to remove the water-soluble substances creatinine and urea and stabilized metabolic dysfunction by increasing pH and negative base excess values. Platelets and fibrinogen levels frequently declined during treatment. Periprocedural bleeding and transfusion of red blood cells were common findings in these patients.

**Conclusions:**

From this clinical perspective ADVOS and OPAL may provide higher reduction capabilities of liver solutes (i.e. bilirubin and ammonia) in comparison to MARS and SPAD. However, further prospective studies comparing the effectiveness of the devices to support liver impairment (i.e. bile acid clearance or albumin binding capacity) as well as markers of renal recovery are warranted.

## Introduction

**Figure 1.**
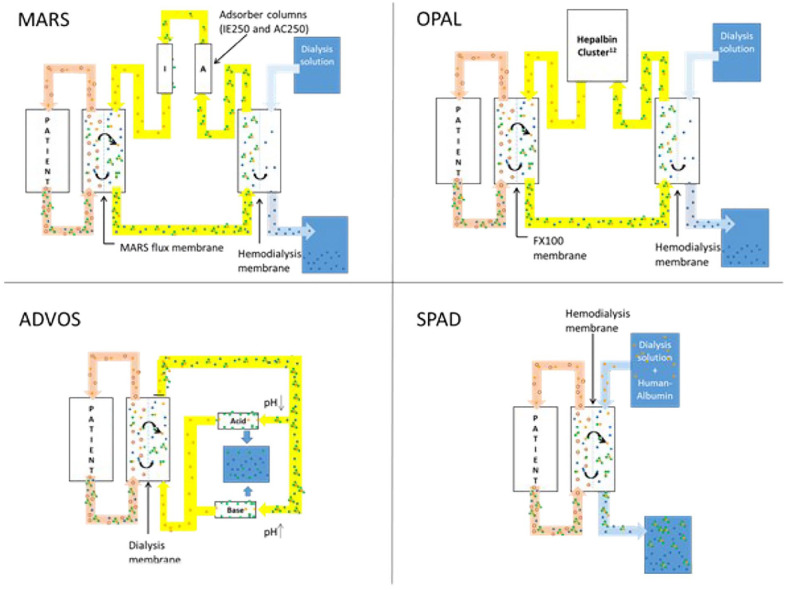
Principles of machine setup among the different ECAD devices. MARS: molecular adsorbent recirculating system; ADVOS: advanced organ support system; SPAD: single pass albumin dialysis; OPAL: open albumin.

Therapeutic options of critically ill patients with liver failure are scarce. Beside standard medical therapy and critical care monitoring, extracorporeal liver support may provide therapeutic options, especially in case of progression to multiorgan failure. Within recent years, several devices performing extracorporeal liver support became available. Based on the underlying concept artificial organ support can be divided into systems using plasma separation (i.e. plasmapheresis or the Prometheus system) or albumin dialysis. Currently, the following extracorporeal albumin dialysis devices (ECAD) were clinically applied: molecular adsorbent recirculating system (MARS), open albumin (OPAL), advanced organ support (ADVOS), and single pass albumin dialysis (SPAD). MARS was invented in 1995 by Mitzner and Stange.^1^ The system consists of two components, MARS dialyzer and conventional hemodialysis machine. Within the system three circuits can be defined: patient blood, albumin and hemodialysis curcuit. Recirculating albumin is regenerated via two adsorber columns, namely AC250 and IE250.^2^ OPAL is based on MARS: However, albumin regenerating columns were replaced by the Hepalbin adsorbant (Hepalbin-Cluster^12^) that are attached to the MARS monitor.^3^ The cluster incorporates powdered activated Hepalbin charcoal on a cellulose framework combined with a cationic binding polymer.^4^ ADVOS uses the aforementioned circuits, incorporated into a separate machine setup, but albumin is regenerated by adding acid and bases.^5^ SPAD represent a rapid and easy to setup ECAD system using conventional hemodialysis machines. Albumin is added to the hemodialysis solution, allowing albumin-bound toxins to pass the hemodialysis filter. In contrast to other systems albumin solution does not recirculate and is wasted after passing the hemodialysis filter.^6^ All systems are depicted in Figure 1 and further described in Table 1.

**Table 1.** Principle mechanism, machine setup, equipment, and costs of the ECAD devices.

	MARS	OPAL	ADVOS	SPAD
Principle mechanism	Albumin circulation in a separate circuit and regeneration via IE250 and AC250 resin columns	Albumin circulation in a separate circuit and regeneration via Hepalbin-cluster^12^	Albumin circulation in a separate circuit and regeneration via acid and base addition	Albumin addition to conventional hemodialysis solution to a final concentration of 4% Wastage of albumin solution after passing the hemodialysis filter
Machine Setup	MARS-Monitor attached to hemodialysis machine	MARS-Monitor attached to hemodialysis machine	ADVOS machine	hemodialysis machine
Albumin content	500 ml 20% human albumin	400 ml 20% human albumin	200 ml 20% human albumin	1000 ml 20% human albumin
Equipment costs, including human albumin	2400 €	2600 €	2900 €	750 €
Treatment duration	8 h	up to 24 h	24 h	7 h

MARS: molecular adsorbent recirculating system; OPAL: open albumin; ADVOS: advanced organ support system; SPAD: single pass albumin dialysis.

All systems were shown to reduce patient’s bilirubin levels—a surrogate for successful albumin dialysis. Moreover, creatinine and urea decline during hemodialysis. Most experience was gained with MARS, showing benefits in acute (ALF) and acute-on-chronic (ACLF) liver failure with respect to hepatic encephalopathy, pruritus, hemodynamic improvement, and hepatorenal syndrome. However, large randomized trials failed to demonstrate survival benefit.^7^

Hence, little is known about detoxification capabilities, efficacy, and efficiency among the devices. We retrospectively evaluated the detoxification capabilities of MARS, OPAL, ADVOS, and SPAD in critically ill patients with either ALF, ACLF, or transplant failure. We hypothesize comparable detoxification and hemodialysis capacities among the ECAD devices.

## Materials and methods

### Study design

**Figure 2.**
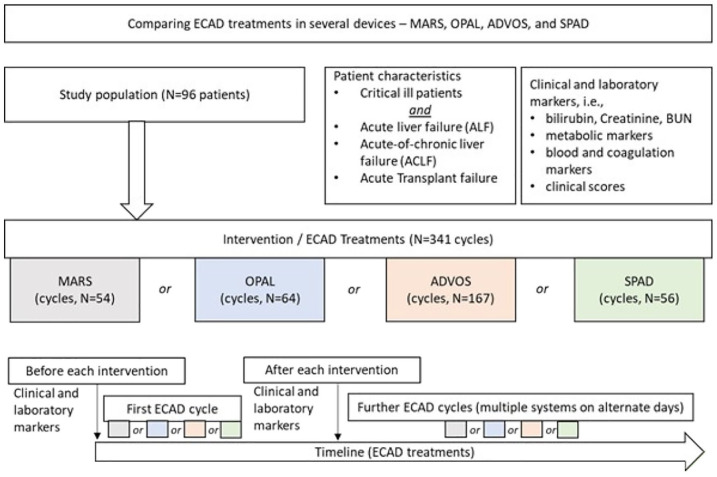
Flowchart depicting the study design and evaluation strategy.

At the interdisciplinary intensive care unit of Jena university hospital, the ECAD devices MARS, SPAD, ADVOS, and OPAL were available and frequently in use. We performed a retrospective single-center data analysis of all ECAD patients between 2015 and 2021 in our institution treated with the aforementioned systems. According to our local standard operating procedure, ECAD is considered in patients with diagnoses of ALF, ACLF, or liver transplant failure presenting with the following signs and symptoms: (1) Plasma disappearance rate of indocyanine green (PDR_ICG_) <8–10%/min, (2) plasma bilirubin levels >170 μmol/l, and (3) international normalized ratio (INR) >1.5 and/or (4) symptoms of hepatic encephalopathy grade II or higher. Patients with a pertinent diagnosis were identified by the intensivist in charge, who schedules ECAD. Selection of the devices were based on availability and patient comorbidities. Hence, since availability of the OPAL system MARS is no longer in use in our institution. SPAD is commonly deployed in case of urgent or unscheduled ECAD, especially during the night or on weekends. Moreover, in case of simultaneous ECAD treatments SPAD is quick and easy to set up in parallel. ADVOS is preferentially initiated in case of concomitant pulmonary or metabolic derangements, while OPAL represents the treatment of choice in all other cases in our institution. Treatments were identified by screening the data management system (SAP, Version 7300.1.3.1079) for Operation and Procedure classification system (OPS) Code 8-858—liver dialysis. Study design and evaluation strategy are depicted in Figure 2. The study was approved by the local ethical committee (5467-03/18 and 2022-2710-Daten).

### Patient characteristics and periprocedural parameters

Clinical data, including age, gender, and diagnosis on admission were recorded. Based on the extracorporeal liver support system, treatments were divided into four groups: MARS, OPAL, ADVOS, and SPAD. Patients treated with several systems on different days were also included in the analysis. Laboratory parameters, health scoring values, and vasopressor requirement (i.e. norepinephrine) before and after each treatment were recorded without correction for hemoconcentration at the end of treatment. Transfusion requirements were recorded during each procedure.

### Settings of extracorporeal liver support systems

MARS (Gambro, Lund, Sweden) was built up according to the manufacturer’s instructions and attached to a standard hemodialysis machine (Edwards BM25; Edwards Life Science, Unterschleissheim, Germany). With MARS the IE250 and AC250 adsorbers were implemented in the MARS monitor, while in OPAL the Hepalbin adsorbant (Hepalbin-Cluster^12^, Albutec GmbH, Rostock, Germany) was attached instead of the adsorbers. In case of MARS the albumin circuit was primed with 600 ml and in OPAL with 500 ml of a 20% human albumin solution (Albutein 20 g/100 ml, Grifols Deutschland GmbH, Germany). ADVOS was set up according to the manufacturer’s instructions. Albumin circuit was primed using 200 ml of 20% albumin solution (Albutein 20 g/100 ml, Grifols Deutschland GmbH, Germany).

For SPAD (multiFiltrate; Fresenius Medical Care, Bad Homburg, Germany), 1 l of fluid was removed from an 5000 ml dialysis solution bag (Ci-Ca Dialysate K2 Plus in case of regional citrate anti-coagulation or multiBic dialysate for heparin anti-coagulation; Fresenius Medical Care) and replaced by 1 l of 20% albumin solution (Albutein 20 g/100 ml, Grifols Deutschland GmbH, Germany) to get an albumin concentration of 4%. Using a 5000 ml dialysis solution bag, flow rate of 700 ml/h resulted in a treatment cycle of about 7 h.^8^ Table 1 and Figure 1 highlight the basic principle, machine setup, and costs of the referring ECAD device.

Vascular access was obtained through double-lumen hemodialysis catheter (Gamcath High Flow Double Lumen Catheter Kit, 13 F, Gambro Kathetertechnik Hechingen, Germany), placed either in femoral or jugular veins. Blood flow rates were set at between 100 and 150 ml/min according to patient’s hemodynamics. Albumin flow rates were set at 200 ml/min in case of MARS or OPAL and 320 ml/min in case of ADVOS, according to manufacturer’s recommendations.

Blood anti-coagulation was maintained either using regional citrate application or by systemic infusion of unfractionated heparin. In case of regional citrate anti-coagulation, citrate (4% sodium citrate; Fresenius Kabi, Bad Homburg, Germany) was applied before the hemofilter aiming a final ionized postfilter calcium level of 0.25–0.45 mmol/l, followed by calcium reversal (1 N calcium chloride solution; Serumwerk Bernburg AG, Bernburg, Germany). In absence of bleeding infusion of unfractionated heparin wase adjusted to an activated clotting time of 140–200 s.

### Statistical analysis

Data are presented as median values [25th–75th percentile] and categorical data as number and percentage, unless otherwise indicated. To compare the effects of ECAD on several parameters we used Generalized Estimating Equations (GEE). This method accounts for correlated data due to repeated measurements of several ECAD cycles and devices per patient. We estimated mean differences between the devices with 95% confidence intervals adjusted for the cycle-specific baseline value of the analyzed parameter and duration of treatment cycle, respectively.

Categorical variables were analyzed by χ^2^ test. With respect to the first treatment cycle group comparisons were evaluated using Wilcoxon rank sum test. A *p*-value <0.05 was considered statistically significant. Statistical analyses were performed using SPSS 27 (IBM SPSS statistics). Figures were designed using SigmaPlot version 14.0 (Systat Software, Erkrath, Germany).

## Results

### Patient characteristics

Between 2015 and 2021 *n* = 341 ECAD cycles in *n* = 96 patients were eligible for the final evaluation, thereof *n* = 54 (15.8%) MARS, *n* = 64 (18.7%) OPAL, *n* = 167 (48.8%) ADVOS, and *n* = 56 (16.4%) SPAD treatments. Patients received in median 3 [1.0–5.0] cycles of ECAD, with a minimum of 1 and a maximum of 17 cycles per patient. Patients had a median age of 54 [43.8–64.0] years and the majority (*n* = 60 (62.5%)) were of male gender. Deterioration of primary liver failure was the leading cause for ECAD in *n* = 71 (74.0%) patients, while *n* = 25 (26.0%) patients were treated with ECAD following secondary liver failure in context of multiorgan failure. On hospital admission patients had a median MELD score of 33 [22.5–38.0], an APACHE-II score of 25 [20.0–29.2], SAPS-II score of 50 [41.8–62.3], and median SOFA score of 12 [9.0–16.0]. There were no differences between within the patient characteristics between the ECAD groups. Table 2 highlights patient characteristics in the total cohort and separated by treatment group. Median MARS treatments lasted for 8 [6.7–9.0] h, according to manufacturer’s recommendations. Median application of OPAL was 21 [13.0–24.0] h. Median duration of ADVOS was 14 [9.0–16.0] h, dependent on wastage of the dialysis concentrate. Technically, one SPAD cycle lasted for 7 h, but was frequently continued as conventional renal replacement therapy. Therefore, median duration of SPAD was 13 [7.0–54.0] h. MARS treatment was preferentially performed using heparin anticoagulation, while citrate was the leading anticoagulation strategy in all other ECAD treatments. The majority of patients (*n* = 70 (73.7%)) died within hospital.

**Table 2.** Patient characteristics on ICU admission.

Characteristics	Total cohort	ADVOS	OPAL	MARS	SPAD	*p*-value
N	96	52	14	13	17	
Age, year						
Median (IQR)	54 [44.0–64.0]	60 [49.5–65.8]	48 [44.8–58.5]	51 [38.0–64.5]	60 [50.0–68.5]	n.s.
Minimum/maximum	20.0/82.0	20.0/82.0	26.0/64.0	28.0/70.0	44.0/76.0	
Male sex, *n* (%)	60 (62.5)	34 (65.4)	7 (50.0)	8 (61.5)	11 (64.7)	n.s.
Outcome, *n* (%)						
ICU mortality	61 (63.5)	37 (71.2)	7 (50.0)	6 (46.2)	11 (64.7)	n.s.
Hospital survival	26 (27.1)	10 (19.2)	5 (35.7)	7 (53.8)	4 (23.5)	
Scores at admission ICU, median (IQR)						
MELD	33 [22.5–38.0]	31 [17.0–40.0]	32 [28.0–35.8]	32 [21.8–35.0]	35 [23.3–40.0]	n.s.
APACHE II	25 [20.0–29.3]	25 [20.0–32.0]	23 [20.5–27.3]	26 [19.5–30.0]	25 [21.3–30.0]	n.s.
SAPS II	50 [41.8–62.3]	50 [43.0–61.0]	50 [39.0–57.8]	49 [39.5–65.0]	52 [41.0–68.3]	n.s.
SOFA	12 [9.0–16.0]	13 [9.0–16.0]	12 [9.8–17.3]	12 [10.5–14.0]	12 [7.0–15.5]	n.s.
RRT during the first 24 h of ICU admission, *n* (%)	56 (58.3)	23 (44.2)	6 (42.9)	6 (46.2)	8 (47.1)	n.s.
Liver failure, *n* (%)						
Primary liver failure	71 (74.0)	40 (76.9)	10 (71.4)	10 (76.9)	11 (64.7)	n.s.
Secondary liver failure	25 (26.0)	12 (23.1)	4 (28.6)	3 (23.1)	6 (35.3)	
Number of treatments						
Median (IQR)	3 [1.0–4.8]	3 [1.0–5.0]	3 [2.8–8.3]	2 [1.0–3.0]	2 [1.0–3.5]	n.s.
Minimum/maximum	1/17	1/17	1/12	1/10	1/10	

ICU: intensive care unit; APACHE II: Acute Physiology and Chronic Health Evaluation II; SAPS II: Simplified Acute Physiology Score II; SOFA: Sequential Organ Failure Assessment; RRT: renal replacement therapy; n.s.: no significance.

### Laboratory parameters and clinical scoring

**Figure 3.**
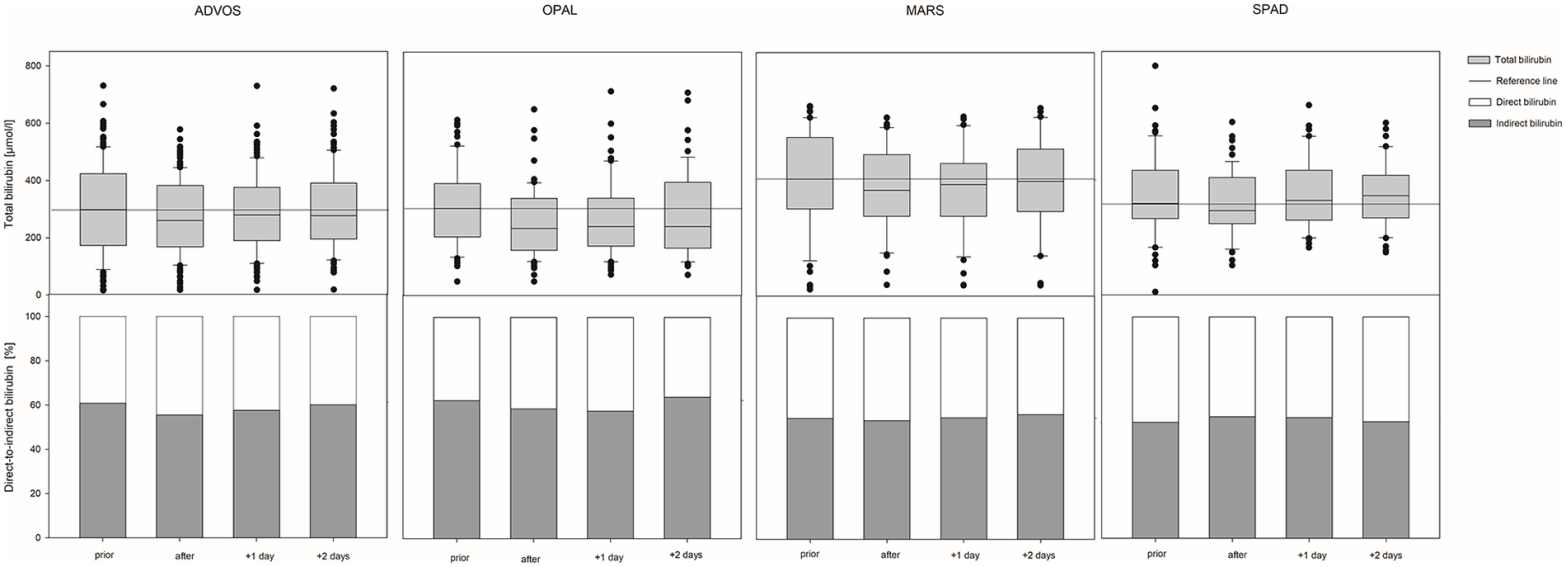
Changes of serum bilirubin and the ratio of direct-to-indirect bilirubin during ECAD with either molecular adsorbent recirculating system (MARS), advanced organ support system (ADVOS), single pass albumin dialysis (SPAD) or open albumin (OPAL).

**Figure 4.**
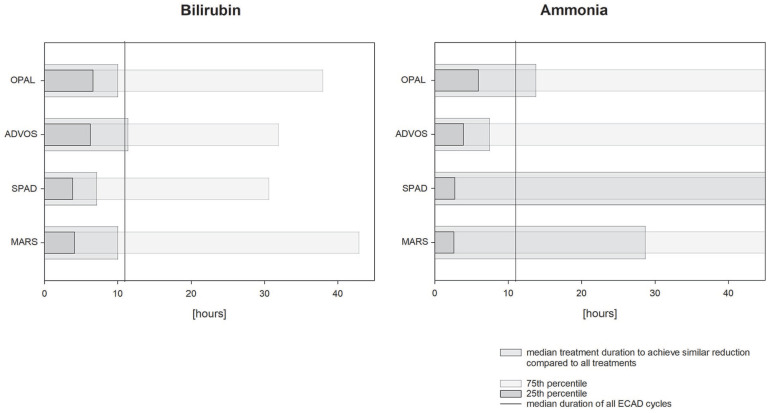
Runtime of each of the ECAD devices in comparison to the median runtime and reduction of bilirubin and ammonia levels. Bars represent median duration and 25th- or 75th percentile of each device to achieve similar bilirubin or ammonia reduction in comparison to the median duration of all ECAD devices (represent by vertical line). MARS: molecular adsorbent recirculating system; ADVOS: advanced organ support system; SPAD: single pass albumin dialysis; OPAL: open albumin.

**Figure 5.**
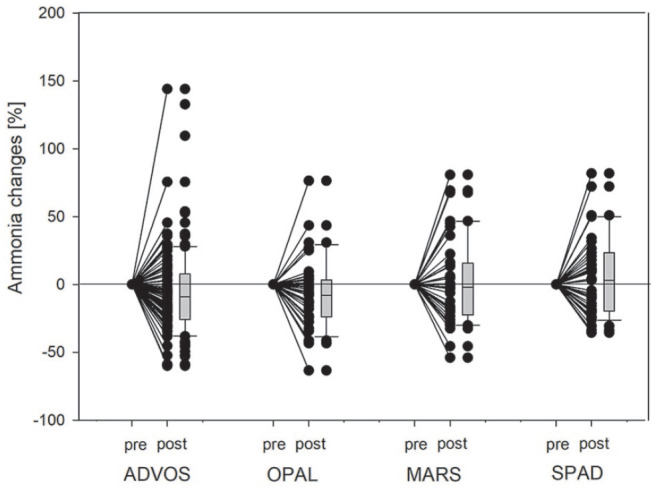
Percental changes of ammonia levels during ECAD with either molecular adsorbent recirculating system (MARS), advanced organ support system (ADVOS), single pass albumin dialysis (SPAD), or open albumin (OPAL). Dots represent changes of ammonia levels after treatment in comparison to initial values in every individual treatment. Boxplots summarize the entire treatment sessions.

ECAD treatment resulted in significant decrease of serum bilirubin levels in each of the devices (see Figure 3), with a median bilirubin reduction of 9.8 [−22.00 to −0.43] % (−31 [−76.0 to −1.8] µmol/l). The duration of all ECAD treatments in median was 11 [8.0–16.0] h and the median percental bilirubin reduction −1.0 [−1.77 to −0.05] % per h. With respect to the referring devices MARS resulted in a median bilirubin reduction of −0.9 [−2.38 to −0.23] % per h, OPAL −0.9 [−1.48 to −0.26] % per h, ADVOS −0.9 [−1.57 to +0.31] % per h, and SPAD −1.3 [−2.53 to −0.32] % per h. To compare the ability of the various devices in reducing patient’s bilirubin levels, the percental reduction per hour of the referring devices were related to the median bilirubin reduction and duration of all devices. Here, MARS would need at least to run for 10 [4.1–42.6] h, OPAL 10 [6.6–37.7] h, ADVOS 11 [6.2–32.6] h, and SPAD 7 [3.9–30.6] h to achieve similar bilirubin reduction (see Figure 4). Bilirubin levels returned to baseline level immediately the day after SPAD treatment, while in the other devices total bilirubin values stayed significantly lower compared to the baseline the day after treatment. Nevertheless, 2 days after treatment total bilirubin level rebounded to baseline in all devices. The ability of removing strong albumin bound bilirubin (interpreted by using the ratio between direct and indirect bilirubin) did not change in either of the devices during treatment (see Figure 3). With respect to ammonia purification, only ADVOS (in median: −6 [−21.0 to +5.8] µmol/l (−9%)) and OPAL (in median: −4 [−21.0 to +1.0] µmol/l (−8%)) were able to significantly alter patient’s ammonia levels, while MARS and SPAD did not (see Figure 5). With respect to the referring devices MARS resulted in a median ammonia reduction of—0.3 [−2.76 to +2.04] % per h, OPAL −0.5 [−1.2 to +0.18] % per h, ADVOS −0.9 [−1.82 to +1.21] % per h, and SPAD +0.4 [−2.58 to +2.01] % per h, while ammonia reduction in all devices were −0.6 [−1.96 to +1.38] % per h. Therefore, to achieve similar ammonia reduction rates MARS would need to last for 29 [2.6–50.] h, OPAL 14 [5.9–50], ADVOS 8 [3.9–50], and SPAD 71 [2.8–50] h (see Figure 4) compared to the median duration of all ECAD devices. Parameters related to kidney function were also significantly altered during treatment. Levels of hemoglobin and hematocrit remained unchanged during therapy, while platelets and fibrinogen levels were significantly lowered. Base excess and pH levels significantly increased during treatment. Lactate values significantly increased but stayed in median within the reference range. Levels of APACHE-II and SAPS-II remained unchanged during treatment, while SOFA scores in median increased by 1 point during ADVOS treatment. Changes in laboratory parameters and clinical scoring for each of the devices are depicted in Supplemental Tables S1a and S1b. Using Generalized Estimating Equations parameters related to kidney function were significantly reduced is SPAD compared to the other systems, while all other evaluated parameters were comparable between the treatment modalities (see Table 3).

**Table 3.** Percental change in laboratory and clinical parameters during ECAD (repeating cycles).

	Total cohort	MARS	ADVOS	SPAD	OPAL	*p*-value
Bilirubin [%]-change	−9.8 [−22.00 to −0.44]	−7.8 [−18.97 to −1.83]	−9.9 [−20.15 to 4.53]	−9.7 [−22.32 to −2.11]	−15.9 [−29.66 to −4.93]	0.06
Ammonia [%]-change	−7.1 [−23.83 to 13.12]	−2.1 [−22.56 to 15.63]	−8.9 [−25.57 to 8.17]	3.0 [−19.75 to 23.40]	−8.0 [−23.76 to 3.23]	0.47
Creatinine [%]-change	−12.3 [−27.47 to 2.53]	−10.7 [−23.49 to 2.70]	−13.0 [−29.23 to 0.00]	−6.8 [−16.21 to 8.99]	−22.4 [−37.39 to −3.85]	0.00
Urea [%]-change	−15.4 [−32.72 to 2.86]	−6.5 [−22.68 to 7.88]	−24.8 [−39.40 to −3.58]	−6.8 [−15.41 to 14.34]	−18.8 [−32.10 to 1.72]	0.00
Hemoglobin [%]-change	−1.4 [−7.14 to 5.99]	−2.1 [−8.43 to 2.06]	−1.8 [−7.46 to 5.88]	0.0 [−5.43 to 8.27]	0.0 [−4.84 to 6.09]	0.64
Platelet count [%]-change	−13.8 [−32.84 to 8.00]	−3.1 [−21.43 to 9.37]	−20.6 [−41.76 to 3.45]	−11.5 [−26.67 to 10.00]	−2.9 [−28.31 to 12.91]	0.99
Fibrinogen [%]-change	−6.9 [−25.45 to 3.57]	−9.2 [−17.76 to 0.00]	−10.0 [−33.33 to 6.67]	−1.2 [−9.48 to 4.44]	−7.0 [−19.88 to 0.00]	0.60
INR [%]-change	0.0 [−7.14 to 12.09]	0.0 [−2.94 to 12.91]	0.0 [−8.89 to 14.29]	0.0 [−5.00 to 8.52]	0.0 [−7.69 to 9.09]	0.63
Lactate [%]-change	3.6 [−14.90 to 30.86]	14.3 [−6.07 to 33.33]	0.0 [−18.90 to 31.01]	8.2 [−7.22 to 37.22]	−5.2 [−19.05 to 18.21]	0.42
pH [%]-change	0.3 [−0.27 to 0.75]	0.3 [−0.27 to 0.68]	0.3 [−0.40 to 0.81]	0.3 [−0.27 to 0.81]	0.3 [−0.14 to 0.67]	0.86
Base excess [%]-change	−25.7 [−80.45 to 39.29]	−21.7 [−52.42 to 36.20]	−42.2 [−111.60 to 19.17]	10.0 [−61.67 to 90.63]	−22.3 [−68.59 to 42.71]	0.07

MARS: molecular adsorbent recirculating system; ADVOS: advanced organ support system; SPAD: single pass albumin dialysis; OPAL: open albumin; INR: international normalized ratio.

First ECAD cycle was associated with the highest percentage reduction in serum bilirubin (in median −65 µmol/l (18%)) and ammonia (in median −11 µmol/l (16.5%)) levels. Moreover, levels of creatinine were also significantly lowered during the first ECAD cycle. All parameters of the first ECAD cycle are shown in Table 4 and in more detail in Supplemental Tables S2a and S2b.

**Table 4.** Percental change in laboratory and clinical parameters during ECAD in first treatment cycle.

	Total cohort	MARS	ADVOS	SPAD	OPAL	*p*-value
Bilirubin [%]-change	−18.0 [−27.18 to −4.97]	−5.0 [−18.16 to 0.00]	−18.5 [−26.43 to −2.49]	−14.3 [−24.21 to −9.14]	−29.1 [−37.97 to −21.62]	0.06
Ammonia [%]-change	−16.4 [−27.38 to 11.98]	10.0 [−22.56 to 32.63]	−17.5 [−30.69 to 2.95]	9.2 [−20.63 to 20.83]	−23.8 [−32.47 to 6.56]	0.172
Creatinine [%]-change	−21.1 [−34.96 to −6.09]	−10.3 [−26.68 to 11.97]	−21.6 [−33.94 to −10.36]	−10.4 [−29.66 to 0.18]	−34.4 [−47.64 to −14.71]	0.022
Urea [%]-change	−23.6 [−41.49 to −10.61]	−12.8 [−23.59 to 7.57]	−38.0 [−48.69 to −19.42]	−9.3 [−18.56 to −0.29]	−18.8 [−33.64 to −9.34]	0.001
Hemoglobin [%]-change	0.0 [−7.48 to 7.28]	−2.0 [−8.23 to 2.50]	−1.8 [−9.80 to 5.88]	7.1 [−2.37 to 13.33]	2.0 [−3.57 to 6.53]	0.108
Platelet count [%]-change	−20.5 [−40.50 to −2.12]	−32.5 [−39.63 to −2.74]	−25.5 [−48.19 to −2.86]	−9.1 [−36.36 to −3.62]	−9.7 [−31.02 to 13.95]	0.292
Fibrinogen [%]-change	−4.8 [−33.33 to 11.43]	−7.3 [−16.67 to 12.86]	−15.6 [−42.33 to 13.84]	0.0 [−31.41 to 8.75]	−8.9 [−25.35 to 9.92]	0.637
INR [%]-change	0.0 [−22.22 to 12.50]	0.0 [−14.29 to 21.15]	0.0 [−22.22 to 12.50]	−5.0 [−21.52 to 8.33]	−4.2 [−25.35 to 10.80]	0.91
Lactate [%]-change	2.6 [−23.49 to 37.65]	1.4 [−25.00 to 23.18]	0.0 [−27.66 to 38.10]	21.7 [−17.03 to 63.92]	−3.2 [−15.50 to 31.55]	0.595
pH [%]-change	0.4 [−0.20 to 0.96]	0.3 [−0.34 to 0.85]	0.5 [0.00–1.33]	0.4 [−0.40 to 0.95]	0.0 [−0.42 to 0.54]	0.085
Base excess [%]-change	−35.9 [−124.14 to 30.23]	−4.1 [−27.78 to 34.83]	−50.0 [−122.35 to 36.25]	−18.6 [−69.84 to 33.71]	−41.7 [−162.66 to 6.24]	0.427

MARS: molecular adsorbent recirculating system; ADVOS: advanced organ support system; SPAD: single pass albumin dialysis; OPAL: open albumin; INR: international normalized ratio.

With reference to heparin and citrate anticoagulation all changes in laboratory and clinical parameters were comparable between the devices. However, lactate values were significantly higher after treatment in SPAD with citrate anticoagulation hinting toward citrate accumulation by applying reduced dialysis flow rates necessary during treatment (see Supplemental Tables S4a, S4b, and S4c).

### Transfusion requirement, bleeding tendency, and adverse events

In *n* = 78 (23%) of ECAD treatments bleeding was noted—mostly due to diffuse bleeding tendency or gastrointestinal bleeding. With respect to the devices, bleeding tendency was more often reported prior (*n* = 42 (25.1%)), during (*n* = 42 (25.1%)), and after (*n* = 39 (23.4%)) ADVOS. Not surprisingly, rates of transfusions of red packed blood, coagulation factors (factor I and prothrombin complex (factor II, VII, IX, and X)) as well as platelet substitution were therefore higher in ADVOS treatments. However, transfusion during ECAD was commonly a rare occasion (details see Supplemental Table S3). Two sessions of ECAD (one MARS and one ADVOS) were preliminary terminated due to filter clotting or urgent liver transplantation. In all other cycles no ECAD related adverse events were reported.

## Discussion

ECAD devices were designed to remove hydrophobic substances accumulating in liver failure.^1^ Currently, four ECAD systems are in clinical use, namely MARS, OPAL, ADVOS, and SPAD. Data on the comparison of the detoxification capacity in all devices are sparse. The results of our retrospective single-center study can be summarized as follows:

1. All devices were effective in removing albumin bound substances, without differences in bilirubin reduction between the devices. However, bilirubin levels returned to baseline values within two days after treatment.
2. Ammonia levels were significantly reduced during ADVOS and OPAL treatments, while MARS and SPAD were not able to significantly reduce ammonia levels.
3. All devices were able to remove water-soluble substances like creatinine and urea and were able to stabilize metabolic dysfunction by increasing pH and negative base excess, without significant differences between the devices.
4. Heparin and citrate anticoagulation resulted in similar changes of laboratory and clinical parameters among the devices. However, lactate significantly increased during SPAD treatment, hinting toward citrate accumulation most likely due to the reduces dialysis flow rates during treatment.
5. Periprocedural bleeding and transfusion of red blood cells were common findings in ECAD patients. However, our data found no increased risk of ECAD treatment to higher bleeding rate during or after application.

In clinical practice decrease in bilirubin levels reflect the surrogate to describe and monitor removal of albumin bound toxins during ECAD. Hence, for all devices successful bilirubin reduction was shown within the literature^8,9^ and also in the current analysis. For all devices comparison of bilirubin removal against MARS is available. Thus SPAD was shown to equally decrease patient’s bilirubin levels in comparison to MARS both in retrospective^8^ and prospective trials.^10,11^ MARS and ADVOS were compared in a retrospective analysis, again without differences with respect to bilirubin levels.^9^ Finally, MARS and OPAL were compared within the OPALESCE trial (EUDAMED (CIV-13-04-010642)). With focus on other liver related solutes, only ADVOS and OPAL led to significant decrease of ammonia levels during treatment, while MARS and SPAD were not able to decrease ammonia. These results are in line with current literature, describing significant reduction of ammonia levels in both experimental and patient settings.^12,13^ Similar results for OPAL were missing so far. For MARS and SPAD no significant changes in ammonia levels were reported.^11,14^ It is therefore not surprisingly that treatment duration to significantly reduce ammonia levels of MARS and SPAD would get unachievable high compared to the median application of all ECAD devices, while for bilirubin all devices show similar application times to achieve similar amounts of bilirubin reduction. From this point of view ADVOS and OPAL seem to be superior over MARS and SPAD in reducing liver solutes. However, with respect to other markers of liver dysfunction MARS was superior over SPAD in removing bile acids and improving albumin binding capacity (ABiC)^10^ and OPAL superior over MARS for both markers (OPALESCE trial). Nevertheless, neither of these parameters are available in clinical routine and the clinical rationale on outcome of these parameters still need to be clarified. This extends to inflammatory markers and cytokine levels. From the pathophysiological point of view cytokines play a pivotal role in liver dysfuntion and progression to multiorgan failure.^15–17^ However, cytokine levels in patients with liver dysfunction and ECAD were low and levels did not significantly change during treatment, either with MARS and SPAD^10^ or ADVOS.^18^

In the current investigation there were no differences between the devices in reducing creatinine and BUN. However, the efficacy of renal replacement therapy may be lower in SPAD in comparison to other devices, as the dialysate flow in SPAD is reduced to allow albumin bound substances to pass the hemodialysis filter into the albumin enriched dialysate. After therapy, dialysate flow can be enhanced using the same set up as used for ECAD. As there were no significant differences in reducing creatinine and urea using either ADVOS^9^ or OPAL in comparison to MARS, SPAD was inferior in reducing creatinine and urea,^8^ due to the reduced dialysis flow rate. However, only for MARS improvement of renal function or hepatorenal syndrome was shown within the literature.^19,20^ Currently, little is known about renal recovery in ECAD patients. Recent knowledge of renal recovery with the emergence of novel biomarkers could be one of the new prognostic tools to solve this issue, but further work is needed in this special patient cohort.

All devices were able to increase pH and negative base excess values. Due to the setup rapid metabolic stabilization is known for ADVOS.^21^ However, especially under citrate anticoagulation rapid stabilization of acidotic states were also possible. Certainly, citrate anticoagulation carries the risk of citrate accumulation, resulting in alkalosis or toxicity, with risk of acidosis. Lactate increase during treatment may hint toward citrate accumulation and should therefore be analyzed carefully. In the current evaluation lactate levels significantly increased during SPAD with citrate anticoagulation. Therefore, critical and careful monitoring of citrate using the ratio of total-to-ionized calcium levels were recommended.^7^ With respect to the current analysis there were no significant differences in metabolic stabilization between the devices. However, we did not analyze the velocity of metabolic stabilization for the single devices.

Bleeding and transfusion were common findings in ECAD patients. Bleeding complications during ECAD vary between 9% and 40% within the literature, mostly resulting from GI-bleeding or from indwelling catheters.^22,23^ In the current analysis bleeding tendency was observed in 23% of cases, most likely due to GI- or diffuse bleeding. Bleeding was more common in ADVOS compared to the other devices. However, as ADVOS presents the most common treatment modality in our analysis and bleeding were already reported prior to treatment, this may present a selection bias in this retrospective analysis. Platelet and fibrinogen drops are also common findings within ECAD.^24^ Therefore, critical monitoring of clinical and laboratory bleeding markers need to be carefully evaluated during ECAD. Moreover, recent consensus statements recommend not to initiate MARS in case of thrombocytopenia (<50 Gpt/l) or fibrinogen levels <1 g/l.^7^

Limitations of the current study include the retrospective character, the monocentric design, and lack of comparable data or literature regarding some of the devices. As ADVOS and OPAL were available only since 2016 and 2015 in our institution, most experience was gained with MARS and SPAD. Another limitation is based on the lack of scientific allocation to either of the ECAD devices. We therefore cannot exclude selection bias. Thus, the results of the study may be interpreted in terms of considerations rather than generalization. However, our study truly reflects the every-day life of this special patient cohort.

## Conclusion

The ECAD devices MARS, OPAL, ADVOS, and SPAD were evaluated with focus on detoxification capacities of serum bilirubin, ammonia, and other clinical and laboratory markers. All devices shared similar bilirubin reduction rates, a surrogate for liver detoxification. On the other hand, ammonia levels were only decreased during ADVOS and OPAL application, while treatment duration of MARS and SPAD would get unacceptable high by trying to achieve similar amounts of ammonia reduction. All devices were comparable with respect to renal replacement therapy. Therefore, with focus on liver related solutes, ADVOS and OPAL may share higher reduction capabilities in comparison to MARS and SPAD. However, further prospective studies comparing the effectiveness of the devices to support liver impairment (i.e. bile acids clearance or ABiC) or markers of renal recovery (i.e. NGAL, HGF, Cystatin C, Proenkephalin A, CCL-14, and TIMP-2/IGFBP-7) are missing. Metabolic derangements could be stabilized with all devices to similar amounts. As patients with liver failure share high bleeding risk, clinical and laboratory coagulation makers should be monitored carefully.

## Supplemental Material

sj-pdf-1-jao-10.1177_03913988231191952 – Supplemental material for Extracorporeal albumin dialysis in critically ill patients with liver failure: Comparison of four different devices—A retrospective analysis Click here for additional data file. Supplemental material, sj-pdf-1-jao-10.1177_03913988231191952 for Extracorporeal albumin dialysis in critically ill patients with liver failure: Comparison of four different devices—A retrospective analysis by Oliver Sommerfeld, Caroline Neumann, Jan Becker, Christian von Loeffelholz, Johannes Roth, Andreas Kortgen, Michael Bauer and Christoph Sponholz in The International Journal of Artificial Organs
