# Supplementary material for: Extracorporeal albumin dialysis in critically ill patients with liver failure: Comparison of four different devices—A retrospective analysis
Source: Int J Artif Organs. 2023 Aug 23;46(8-9):481–91. doi: 10.1177/03913988231191952 (PMC10483887; doi:10.1177/03913988231191952)
Supplement: sj-pdf-1-jao-10.1177_03913988231191952 – Supplemental material for Extracorporeal albumin dialysis in critically ill patients with liver failure: Comparison of four different devices—A retrospective analysis [file sj-pdf-1-jao-10.1177_03913988231191952.pdf]

Extracorporeal albumin dialysis in critically ill patients with liver failure: comparison four different devices– a retrospective analysis

-Supplemental data -

Oliver Sommerfeld; Caroline Neumann; Jan Becker Christian von Loeffelholz; Johannes Roth; Andreas Kortgen; Michael Bauer; Christoph Sponholz

Friedrich-Schiller-University Jena, Jena University Hospital, Department of Anaesthesiology and Critical Care Medicine, Jena, Germany

Corresponding author

Oliver Sommerfeld
Friedrich-Schiller-University Jena,
Jena University Hospital,
Department of Anaesthesiology and Critical Care Medicine,
Am Klinikum 1
07747 Jena, Germany
Tel.: +49 – 3641 – 9323294
Fax: +49 – 3641 – 9323102
eMail: oliver.sommerfeld@med.uni-jena.de

Table S1a: Changes in laboratory markers and clinical scorings during ECAD treatment in MARS and ADVOS

		pre-MARS	post-MARS	p-value	pre-ADVOS	post-ADVOS	p-value
Bilirubin	[µmol/l]	389 [303,8 - 554,0]	369 [278,0 - 494,0]	**0.001**	298 [172,0 - 424,0]	261,0 [168,5 - 382,0]	**0.001**
AST	[µmol/l.s]	2,03 [1,045 - 3,900]	1,93 [1,073 - 3,805]	0.992	3,23 [1,450 - 12,900]	3,12 [1,600 - 11,900]	**0.007**
ALT	[µmol/l.s]	1,13 [0,575 - 1,770]	1,04 [0,570 - 2,060]	0.543	1,86 [0,665 - 9,963]	1,79 [0,825 - 8,610]	**0.001**
GLDH	[nmol/l.s]	84 [50,0 - 329,5]	89 [50,0 - 336,5]	0.469	140 [50,0 - 1621,0]	129 [50,0 - 1275,5]	**0.001**
µ-GT	[µmol/l.s]	1,08 [0,568 - 4,083]	1,07 [0,495 - 4,490]	**0.005**	2,44 [0,880 - 3,930]	2,10 [0,900 - 3,900]	0.375
AP	[µmol/l.s]	2,35 [1,628 - 9,470]	2,62 [1,570 - 7,630]	0.107	2,87 [1,755 - 6,795]	2,94 [1,528 - 6,993]	0.365
Creatinine	[µmol/l]	113 [68,8 - 153,0]	83 [61,8 - 160,5]	**0.001**	141 [82,0 - 215,0]	117 [72,5 - 177,0]	**0.001**
Urea	[mmol/l]	16,0 [10,28 - 22,25]	16,2 [10,90 - 21,03]	0.107	11,4 [6,60 - 17,10]	9,1 [5,30 - 12,95]	**0.001**
Hemoglobin	[mmol/l]	5,0 [4,80 - 5,40]	5,0 [4,70 - 5,15]	**0.046**	5,3 [4,90 - 5,70]	5,3 [4,80 - 5,60]	0.166
Hematocrite	[%]	0,24 [0,230 - 0,260]	0,24 [0,223 - 0,250]	0.134	0,25 [0,230 - 0,280]	0,25 [0,230 - 0,270]	0.142
Platelet count	[Gpt/l]	59 [30,8 - 98,3]	59 [22,3 - 87,8]	0.126	55 [36,8 - 102,0]	51 [28,0 - 78,0]	**0.001**
Fibrinogen	[g/l]	2,1 [1,25 - 3,20]	1,9 [1,00 - 3,15]	**0.001**	1,5 [0,90 - 2,50]	1,5 [0,70 - 2,30]	**0.001**
pTT	[sec.]	47,9 [41,28 - 57,55]	47,9 [40,10 - 60,95]	0.095	50,0 [42,00 - 66,00]	52,0 [44,00 - 69,50]	**0.005**
Lactate	[mmol/l]	2,1 [1,38 - 3,40]	2,3 [1,40 - 4,25]	**0.003**	2,3 [1,60 - 5,00]	2,3 [1,60 - 5,00]	0.520
pH		7,35 [7,320 - 7,409]	7,38 [7,340 - 7,422]	**0.006**	7,37 [7,320 - 7,420]	7,39 [7,340 - 7,430]	**0.002**
CRP	[mg/l]	93,1 [58,13 - 121,25]	87,2 [59,58 - 119,50]	0.097	63,3 [29,10 - 115,00]	63,3 [28,85 - 113,00]	0.220
APACHE-II		22 [20,0 - 26,0]	21 [18,0 - 25,5]	0.405	24 [19,0 - 29,0]	24 [19,0 - 30,0]	0.735
SAPS-II		54 [44,5 - 62,0]	50 [45,0 - 62,5]	0.607	52 [43,0 - 67,0]	53 [43,0 - 70,0]	0.044
SOFA		14 [11,8 - 16,3]	13 [12,0 - 16,5]	0.215	15 [13,0 - 18,0]	16 [14,0 - 19,0]	**0.001**

MARS: Molecular Adsorbent Recirculating System; ADVOS: Advanced Organ Support System

AST: Aspartate-Amino-Transferase; ALT: Alanine-Amino-Transferase; GLDH: Glutamatedehydrogenase; µ-GT: gamma-Glutamyl-Transferase

pTT: Partial Thromboplastin Time; CRP: C-Reactive Protein

APACHE: Acute Physiology and Chronic Health Evaluation; SAPS: Simplified Acute Physiologic Score; SOFA: Sequential Organ Failure Assessment

Table S1b: Changes in laboratory markers and clinical scorings during ECAD treatment in SPAD and OPAL

		pre-SPAD		post-SPAD		p-value	pre-OPAL		post-OPAL		p-value
Bilirubin	[µmol/l]	320	[267,0 - 435,5]	295	[249,3 - 410,0]	**0.001**	304	[204,8 - 395,5]	234	[158,5 - 339,3]	**0.001**
AST	[µmol/l.s]	2,59	[1,895 - 6,543]	2,42	[1,815 - 7,483]	**0.006**	1,59	[1,220 - 2,020]	1,60	[1,200 - 1,875]	**0.040**
ALT	[µmol/l.s]	2,21	[1,198 - 2,945]	2,04	[1,245 - 3,843]	**0.014**	0,89	[0,348 - 2,123]	0,90	[0,500 - 2,050]	0.112
GLDH	[nmol/l.s]	226	[77,3 - 934,3]	164	[73,8 - 937,0]	**0.040**	1	[0,0 - 50,0]	1	[0,0 - 50,0]	0.019
µ-GT	[µmol/l.s]	1,82	[1,028 - 4,480]	1,73	[0,895 - 3,628]	0.346	0,98	[0,500 - 2,180]	1,00	[0,500 - 2,400]	**0.001**
AP	[µmol/l.s]	3,03	[2,023 - 6,380]	3,13	[2,000 - 6,225]	**0.021**	2,08	[1,285 - 6,220]	1,90	[1,273 - 5,883]	0.732
Creatinine	[µmol/l]	145	[84,8 - 204,5]	139	[105,5 - 193,0]	0.067	115	[67,5 - 149,0]	77	[52,5 - 121,8]	**0.001**
Urea	[mmol/l]	14,2	[7,35 - 20,45]	15,0	[10,55 - 19,43]	0.297	12,2	[7,23 - 16,30]	10,4	[6,40 - 13,10]	**0.001**
Hemoglobin	[mmol/l]	5,2	[4,83 - 5,58]	5,2	[4,83 - 5,58]	0.636	5,0	[4,80 - 5,40]	5,1	[4,73 - 5,30]	0.856
Hematocrite	[%]	0,25	[0,230 - 0,270]	0,24	[0,230 - 0,278]	0.559	0,24	[0,220 - 0,260]	0,24	[0,230 - 0,260]	0.340
Platelet count	[Gpt/l]	58	[41,0 - 82,8]	57	[27,3 - 76,3]	**0.011**	48	[29,0 - 80,5]	41	[26,5 - 83,5]	**0.051**
Fibrinogen	[g/l]	1,8	[0,95 - 3,85]	1,8	[1,03 - 3,78]	0.308	1,7	[1,10 - 3,35]	1,6	[0,90 - 3,40]	**0.009**
pTT	[sec.]	42,3	[36,53 - 53,20]	46,6	[37,83 - 67,35]	**0.001**	45,5	[36,48 - 54,53]	45,3	[34,25 - 58,75]	0.431
Lactate	[mmol/l]	2,3	[1,33 - 4,38]	2,3	[1,50 - 6,08]	**0.004**	2,4	[1,33 - 3,40]	2,4	[1,60 - 3,20]	0.963
pH		7,39	[7,318 - 7,420]	7,40	[7,335 - 7,440]	**0.033**	7,42	[7,383 - 7,445]	7,45	[7,383 - 7,470]	**0.020**
CRP	[mg/l]	79,0	[36,58 - 126,75]	78,8	[44,70 - 135,50]	0.866	73,2	[38,58 - 103,50]	69,9	[40,03 - 96,90]	0.527
APACHE-II		24	[20,0 - 28,0]	24	[20,0 - 27,3]	0.898	19	[17,0 - 24,8]	19	[17,0 - 22,8]	0.503
SAPS-II		59	[47,0 - 69,0]	58	[47,0 - 68,5]	0.935	48	[41,0 - 55,8]	49	[41,0 - 56,0]	0.831
SOFA		14	[12,0 - 17,0]	14	[12,0 - 16,3]	0.569	14	[11,0 - 15,0]	14	[11,0 - 17,0]	0.919

SPAD: Single Pass Albumin Dialysis; OPAL: Open Albumin

AST: Aspartate-Amino-Transferase; ALT: Alanine-Amino-Transferase; GLDH: Glutamatedehydrogenase; µ-GT: gamma-Glutamyl-Transferase

pTT: Partial Thromboplastin Time; CRP: C-Reactive Protein

APACHE: Acute Physiology and Chronic Health Evaluation; SAPS: Simplified Acute Physiologic Score; SOFA: Sequential Organ Failure Assessment

Table S2a: Changes in laboratory markers and clinical scorings during ECAD treatment in MARS and ADVOS with respect to the first treatment cycle

		pre-MARS	post-MARS	p-value	pre-ADVOS	post-ADVOS	p-value
Bilirubin	[µmol/l]	379 [95,0 - 599,5]	360 [174,0 - 583,0]	**0.019**	301 [133,0 - 487,0]	234 [138,3 - 389,0]	**0.001**
AST	[µmol/l.s]	2,29 [1,100 - 9,125]	1,96 [0,950 - 3,850]	0.656	3,48 [1,720 - 27,300]	4,76 [2,000 - 24,450]	0.285
ALT	[µmol/l.s]	1,32 [0,620 - 5,525]	0,83 [0,530 - 1,560]	0.100	1,96 [0,513 - 18,825]	1,60 [0,700 - 10,500]	0.129
GLDH	[nmol/l.s]	103 [50,5 - 1444,5]	75 [50,0 - 786,8]	0.575	169 [51,3 - 4069,3]	158 [59,0 - 2796,0]	0.218
µ-GT	[µmol/l.s]	0,81 [0,660 - 5,075]	1,17 [0,498 - 5,773]	**0.022**	2,54 [0,785 - 4,480]	2,10 [0,700 - 3,900]	**0.026**
AP	[µmol/l.s]	4,21 [1,825 - 14,600]	2,90 [1,668 - 12,750]	0.092	2,86 [1,765 - 6,223]	2,60 [1,483 - 7,088]	0.550
Creatinine	[µmol/l]	115 [85,5 - 219,0]	120 [69,0 - 332,0]	0.286	167 [90,3 - 235,3]	121 [83,5 - 173,8]	**0.001**
Urea	[mmol/l]	14,6 [7,00 - 20,95]	13,3 [11,10 - 16,90]	0.182	13,2 [7,48 - 22,13]	7,7 [5,23 - 14,93]	**0.001**
Hemoglobin	[mmol/l]	5,1 [4,90 - 6,25]	5,1 [4,90 - 5,75]	0.201	5,4 [4,90 - 6,08]	5,4 [4,80 - 5,60]	0.159
Hematocrite	[%]	0,25 [0,230 - 0,315]	0,24 [0,230 - 0,290]	0.153	0,26 [0,230 - 0,298]	0,25 [0,230 - 0,270]	**0.019**
Platelet count	[Gpt/l]	75 [36,5 - 117,0]	51 [19,5 - 88,8]	**0.006**	66 [39,3 - 132,8]	54 [29,0 - 121,0]	**0.001**
Fibrinogen	[g/l]	1,7 [1,00 - 3,25]	2,1 [1,05 - 3,38]	0.581	1,5 [0,70 - 2,43]	1,3 [0,73 - 2,20]	0.049
pTT	[sec.]	48,0 [42,70 - 60,15]	50,8 [38,23 - 61,23]	0.784	53,0 [43,00 - 80,30]	53,6 [44,20 - 79,35]	0.502
Lactate	[mmol/l]	2,7 [1,90 - 9,35]	2,4 [1,45 - 7,48]	0.624	3,1 [1,80 - 11,83]	3,5 [2,00 - 9,10]	0.417
pH		7,36 [7,310 - 7,435]	7,40 [7,343 - 7,428]	0.223	7,33 [7,270 - 7,390]	7,38 [7,333 - 7,430]	**0.001**
CRP	[mg/l]	67,1 [25,10 - 140,00]	79,3 [33,40 - 174,00]	0.859	61,4 [25,20 - 112,75]	60,3 [29,68 - 107,50]	0.312
APACHE-II		23 [20,0 - 27,0]	24 [18,5 - 25,0]	0.196	26 [23,0 - 30,0]	25 [21,0 - 30,0]	0.834
SAPS-II		54 [44,5 - 62,0]	47 [42,0 - 56,0]	0.168	55 [49,0 - 67,0]	59 [48,8 - 75,5]	0.060
SOFA		13 [11,0 - 18,5]	14 [9,8 - 18,3]	0.330	15 [12,0 - 18,8]	16 [14,0 - 19,0]	**0.004**

MARS: Molecular Adsorbent Recirculating System; ADVOS: Advanced Organ Support System

AST: Aspartate-Amino-Transferase; ALT: Alanine-Amino-Transferase; GLDH: Glutamatedehydrogenase; µ-GT: gamma-Glutamyl-Transferase

pTT: Partial Thromboplastin Time; CRP: C-Reactive Protein

APACHE: Acute Physiology and Chronic Health Evaluation; SAPS: Simplified Acute Physiologic Score; SOFA: Sequential Organ Failure Assessment

Table S2b: Changes in laboratory markers and clinical scorings during ECAD treatment in SPAD and OPAL with respect to the first treatment cycle

		pre-SPAD	post-SPAD	p-value	pre-OPAL	post-OPAL	p-value
Bilirubin	[µmol/l]	319 [205,5 - 521,0]	279 [189,3 - 420,5]	**0.004**	335 [268,0 - 479,3]	273 [189,8 - 332,8]	**0.001**
AST	[µmol/l.s]	2,51 [1,665 - 18,575]	2,32 [1,460 - 17,800]	0.06	1,61 [1,190 - 2,825]	1,60 [1,100 - 2,900]	0.401
ALT	[µmol/l.s]	1,09 [0,780 - 9,425]	1,26 [0,790 - 14,100]	0.096	0,78 [0,313 - 2,328]	0,75 [0,300 - 2,150]	0.187
GLDH	[nmol/l.s]	516 [81,0 - 1857,0]	315 [79,0 - 2219,0]	0.038	50 [0,5 - 84,5]	50 [0,0 - 78,3]	0.173
µ-GT	[µmol/l.s]	1,92 [1,060 - 4,085]	1,64 [0,910 - 3,090]	0.532	1,10 [0,650 - 6,325]	1,85 [0,575 - 5,210]	0.754
AP	[µmol/l.s]	2,79 [1,355 - 6,210]	3,28 [1,905 - 7,753]	0.507	2,42 [1,690 - 5,383]	2,51 [1,480 - 6,813]	0.753
Creatinine	[µmol/l]	160 [110,0 - 245,5]	140 [96,0 - 223,0]	**0.012**	143 [112,8 - 221,0]	103 [74,0 - 129,5]	**0.004**
Urea	[mmol/l]	14,8 [6,30 - 20,60]	14,9 [7,13 - 18,28]	0.059	19,9 [9,08 - 24,65]	13,3 [10,93 - 20,13]	**0.008**
Hemoglobin	[mmol/l]	5,1 [4,85 - 5,70]	5,5 [5,10 - 6,20]	0.192	5,1 [4,70 - 5,45]	5,1 [4,68 - 5,40]	0.448
Hematocrite	[%]	0,25 [0,225 - 0,285]	0,27 [0,235 - 0,305]	0.259	0,23 [0,218 - 0,263]	0,24 [0,220 - 0,260]	0.242
Platelet count	[Gpt/l]	61 [44,5 - 99,0]	60 [22,0 - 82,0]	**0.005**	74 [33,0 - 136,0]	58 [35,8 - 107,5]	0.158
Fibrinogen	[g/l]	1,6 [1,13 - 2,38]	1,3 [0,70 - 2,20]	0.789	1,8 [1,1000 - 3,05]	2,0 [0,40 - 4,15]	0.277
pTT	[sec.]	44,9 [35,75 - 65,75]	48,9 [36,20 - 84,00]	0.136	45,5 [41,68 - 55,98]	48,1 [35,25 - 66,48]	0.600
Lactate	[mmol/l]	2,4 [1,50 - 4,15]	2,7 [1,65 - 6,30]	0.061	2,2 [1,30 - 3,43]	2,1 [1,48 - 3,43]	0.875
pH		7,36 [7,280 - 7,420]	7,39 [7,290 - 7,460]	0.609	7,43 [7,378 - 7,442]	7,42 [7,345 - 7,463]	0.969
CRP	[mg/l]	72,2 [30,85 - 85,80]	65,5 [32,70 - 99,80]	0.820	110,0 [29,40 - 148,50]	98,9 [26,10 - 135,00]	0.300
APACHE-II		25 [18,3 - 29,5]	22 [17,5 - 31,5]	0.166	19 [18,0 - 25,8]	19 [16,8 - 21,0]	**0.027**
SAPS-II		61 [44,8 - 78,5]	61 [42,5 - 69,0]	**0.026**	47 [38,3 - 55,3]	47 [41,8 - 58,0]	0.783
SOFA		15 [9,5 - 17,8]	12 [8,0 - 15,5]	**0.011**	14 [10,5 - 15,3]	13 [11,0 - 17,3]	0.804

SPAD: Single Pass Albumin Dialysis; OPAL: Open Albumin

AST: Aspartate-Amino-Transferase; ALT: Alanine-Amino-Transferase; GLDH: Glutamatedehydrogenase; µ-GT: gamma-Glutamyl-Transferase

pTT: Partial Thromboplastin Time; CRP: C-Reactive Protein

APACHE: Acute Physiology and Chronic Health Evaluation; SAPS: Simplified Acute Physiologic Score; SOFA: Sequential Organ Failure Assessment

Table S3: Transfusion requirements during ECAD.

		MARS		ADVOS		SPAD		OPAL	
Red blood cells	[Units]	0	[0-1]	1	[0-2]	0	[0-1]	0	[0-1]
Platelets	[Units]	0	[0-0]	0	[0-0]	0	[0-0]	0	[0-0]
Fresh frozen plasma	[Units]	0	[0-0]	0	[0-0]	0	[0-0]	0	[0-0]
Prothrombin Complex	[IU]	0	[0-0]	0	[0-0]	0	[0-0]	0	[0-0]
Fibrinogen	[g]	0	[0-0]	0	[0-0]	0	[0-0]	0	[0-0]

IU: International Units; g: Grams;

MARS: Molecular Adsorbent Recirculating System
ADVOS: Advances Organ Support System

SPAD: Single Pass Albumin Dialysis
OPAL: Open Albumin

Table S4 a:

		Total cohort								
		Heparin				Citrate				p-value
Bilirubin	[%]-change	-10.4 [	-21.97 -	-3.86]		-9.7 [	-21.77 -	0.00]		0.30
Ammonia	[%]-change	-8.4 [	-23.24 -	10.92]		-7.4 [	-24.12 -	9.88]		0.83
Creatinine	[%]-change	-10.9 [	-27.01 -	1.51]		-13.5 [	-28.85 -	0.14]		0.49
Urea	[%]-change	-5.5 [	-23.33 -	9.13]		-19.6 [	-35.53 -	-0.92]		0.06
Hemoglobin	[%]-change	-2.0 [	-8.16 -	6.00]		0.0 [	-6.52 -	5.90]		0.353.
Platelet count	[%]-change	-9.8 [	-30.33 -	10.49]		-13.4 [	-32.28 -	8.19]		0.48
Fibrinogen	[%]-change	-4.8 [	-15.00 -	0.00]		-8.0 [	-30.79 -	5.48]		0.47
INR	[%]-change	0.0 [	-7.14 -	7.14]		0.0 [	-7.69 -	14.29]		0.15
Lactate	[%]-change	3.6 [	-11.11 -	25.00]		3.9 [	-15.38 -	34.29]		0.94
pH	[%]-change	0.1 [	-0.27 -	0.68]		0.3 [	-0.27 -	0.81]		0.18
Base Excess	[%]-change	-18.6 [	-50.00 -	35.81]		-34.0 [	-111.06 -	44.62]		0.06

INR: International normalized ratio

Table S4b:

		MARS			OPAL		
		Heparin	Citrate	p-value	Heparin	Citrate	p-value
Bilirubin	[%]-change	-8.7 [-21.70 - -4.39]	-7.9 [-15.83 - -4.28]	0.65	-10.8 [-21.14 - -6.85]	-15.9 [-29.70 - -4.62]	0.76
Ammonia	[%]-change	-3.2 [-22.56 - 13.56]	-2.0 [-26.42 - 43.86]	0.74	-8.3 [-23.21 -]	-10.1 [-23.91 - 1.06]	0.71
Creatinine	[%]-change	-11.1 [-25.30 - -3.03]	-9.4 [-19.55 - 12.75]	0.26	-20.1 [-31.19 - 1.65]	-22.4 [-38.89 - -3.85]	0.63
Urea	[%]-change	1.7 [-12.84 - 8.92]	-19.3 [-27.79 - -4.53]	0.12	-7.0 [-20.13 - 3.07]	-19.1 [-32.18 - 2.29]	0.42
Hemoglobin	[%]-change	-2.1 [-7.41 - 2.00]	-2.1 [-9.59 - 2.69]	0.96	9.4 [6.98 - 16.75]	0.0 [-4.92 - 5.88]	0.06
Platelet count	[%]-change	-0.7 [-21.43 - 10.63]	-5.7 [-17.73 - 9.09]	0.77	-2.6 [-23.32 - 43.61]	-2.5 [-27.54 - 13.51]	0.84
Fibrinogen	[%]-change	-5.7 [-12.50 - -3.15]	-16.7 [-46.15 - 1.07]	0.07	-1.1 [-3.66 - 4.69]	-7.7 [-20.00 - 0.00]	0.22
INR	[%]-change	0.0 [-7.14 - 0.00]	9.4 [0.00 - 41.17]	0.06	0.0 [-5.77 - 3.95]	0.0 [-8.33 - 9.09]	0.80
Lactate	[%]-change	11.1 [-2.94 - 31.53]	21.9 [-8.53 - 63.89]	0.37	9.8 [-3.69 - 21.98]	-6.3 [-20.00 - 19.51]	0.32
pH	[%]-change	0.3 [-0.20 - 0.68]	0.1 [-0.27 - 0.50]	0.58	-0.3 [-0.77 - 1.01]	0.3 [-0.13 - 0.67]	0.37
Base Excess	[%]-change	-14.1 [-38.23 - 34.34]	-24.7 [-77.72 - 71.36]	0.52	-40.4 [-174.81 - 2172.12]	-18.6 [-66.67 - 45.45]	0.86

MARS: Molecular Adsorbent Recirculating System; #OPAL: Open Albumin

INR: International normalized ratio

Table S4c:

		ADVOS			SPAD		
		Heparin	Citrate	p-value	Heparin	Citrate	p-value
Bilirubin	[%]-change	-15.0 [-23.65 - -6.25]	-15.9 [-29.70 - -4.62]	0.08	-7.4 [-17.66 - -2.11]	-9.2 [-14.33 - 1.51]	0.70
Ammonia	[%]-change	-10.1 [-22.79 - 8.17]	-10.1 [-23.91 - 1.06]	0.83	-12.4 [-22.80 - 11.48]	3.0 [-16.57 - 26.24]	0.21
Creatinine	[%]-change	-15.2 [-32.25 - -0.41]	-22.4 [-38.89 - -3.85]	0.55	-1.6 [-14.64 - 15.80]	-7.8 [-16.62 - 6.28]	0.48
Urea	[%]-change	-20.0 [-34.45 - 3.80]	-19.1 [-32.18 - 2.29]	0.36	3.6 [-15.25 - 27.67]	-7.6 [-16.32 - 6.90]	0.23
Hemoglobin	[%]-change	-2.8 [-10.54 - 5.43]	0.0 [-4.92 - 5.88]	0.55	-2.4 [-8.38 - 7.14]	3.8 [-2.46 - 8.13]	0.15
Platelet count	[%]-change	-27.6 [-44.28 - -9.50]	-2.5 [-27.54 - 13.51]	0.28	-4.4 [-19.77 - 26.68]	-13.9 [-20.36 - 1.52]	0.25
Fibrinogen	[%]-change	-18.1 [-25.28 - -0.74]	-7.7 [-20.00 - 0.00]	0.54	-2.6 [-8.85 - 7.08]	0.0 [-8.51 - 5.26]	0.90
INR	[%]-change	0.0 [-7.14 - 11.10]	0.0 [-8.33 - 9.09]	0.95	0.0 [-6.47 - 7.14]	0.0 [0.00 - 8.88]	0.17
Lactate	[%]-change	1.8 [-17.08 - 23.44]	-6.3 [-20.00 - 19.51]	0.86	-2.6 [-16.67 - 10.79]	17.0 [-7.14 - 59.17]	**0.04**
pH	[%]-change	0.1 [-0.25 - 0.81]	0.3 [-0.13 - 0.67]	0.76	-0.2 [-0.40 - 0.12]	0.7 [0.00 - 0.88]	**0.00**
Base Excess	[%]-change	-12.2 [-48.55 - 23.27]	-18.6 [-66.67 - 45.45]	0.06	-20.0 [-69.20 - 45.48]	42.1 [-72.17 - 150.18]	0.16

ADVOS: Advanced Organ Support System; SPAD: Single Pass Albumin Dialysis

INR: International normalized ratio
